# Sports participation in sport clubs, gyms or public spaces: How users of different sports settings differ in their motivations, goals, and sports frequency

**DOI:** 10.1371/journal.pone.0205198

**Published:** 2018-10-08

**Authors:** Ineke Deelen, Dick Ettema, Carlijn B. M. Kamphuis

**Affiliations:** 1 Department of Human Geography and Spatial Planning, Faculty of Geosciences, Utrecht University, Utrecht, The Netherlands; 2 Department of Interdisciplinary Social Science, Faculty of Social and Behavioural Sciences, Utrecht University, Utrecht, The Netherlands; Sao Paulo State University - UNESP, BRAZIL

## Abstract

**Background:**

To develop targeted policy strategies to increase sports participation, more insight is needed into the behavioural patterns and preferences of users of different club-organized (i.e., sports clubs) and non-club organized (i.e., gyms, health centres or swimming pools) or informal sports settings such as public spaces. This study investigates 1) how users of different settings differ regarding self-determined motivations and goals, and sociodemographic and sports-related characteristics, and 2) how the association of motivations and goals with sports participation may differ between users of different sports settings.

**Methods:**

Data were collected through online surveys among Dutch adults aged 18–80 years (N = 910). Ordinal regression analyses were used to investigate the effects of sports settings, the level of self-determined motivations and goals, and interaction effects of motivations and goals with different sports settings, on sports frequency.

**Results:**

Users of different sports settings differed in their personal characteristics, motivations and goals. In general, controlled motivations were negatively associated with sports frequency (B = -0.46). However, among club members, extrinsic goals related to image (B = 0.44), as well as intrinsic goals related to skill development (B = 0.40) and social affiliation (B = 0.47) had significant positive associations with sports frequency. Health-related goals significantly increased sports frequency among users of informal settings, such as public spaces.

**Conclusion:**

The association of motivational variables with sports participation differs between settings. This implies that sports frequency is higher when participants engage in settings that better fit their motivations and goals. Because of the growing importance of informal and flexible settings and health goals, professionals in the sports and health domains should take into account the motivations, goals and needs of different target groups who (want to) use unorganized, informal sports settings including public spaces.

## Introduction

Increasing participation in sports and physical activity is an important health objective in developed countries [1,2]. An important way for (local) governments to achieve this objective is to provide easily accessible facilities where sports can be practised. While the term ‘facilities’ traditionally referred to indoor or outdoor public facilities for specific types of sports, often facilitating voluntary sports clubs, it currently refers to a wide spectrum of settings. Recently, several new opportunities to practice sports have emerged, and especially informal and flexible types of sports participation (also referred to as ‘light’ sports settings) have increased more rapidly than traditional organized club-based sports participation (or ‘heavy’ sports settings) [2–5].

Typical informal and flexible sports settings are commercial health centres and gyms, informal groups and individual participation in the public space, all of which make participants less dependent on formal structures such as membership obligations, opening hours and the availability of specific sports facilities [6,7]. Informal, unorganized and individual types of sports such as running, cycling, and working out in the gym have become increasingly popular, which has resulted in a greater variety of geographical locations used for sports activities, including public spaces and natural environments [3,8,9]. According to Borgers et al. [6], these changes in sports participation can be seen as an issue of institutional change, which is related to processes of cultural and societal change and changing values, habits and attitudes of sports participants. In this paper we build upon definitions of sports participation used in the previous literature [10–12] and we distinguish between the following sports settings: 1) club-organized sports settings (i.e., voluntary sports clubs), 2) non-club organized settings (i.e., gyms, health centres or swimming pools) and 3) informal settings such as public spaces.

To develop targeted policy strategies to increase sports participation levels, more insight into the behavioural patterns and preferences of users of different sports settings is needed. Some studies suggest that preferences for specific sports settings depend on sociodemographic characteristics. For instance, women are more likely to engage in informal and flexible sports in commercial or alternative settings [2,4,8], and adults of higher social classes and with higher incomes are more likely to engage in non-organized sports [13,14]. In addition, Borgers et al. [5] have found that participation frequency and time spent on sports is higher among members of sports clubs in certain types of sports, in contrast to the frequency of engagement of non-organized sports participants.

Previous studies have shown that a range of different factors is associated with sports participation, including sports frequency [5,15,16]. In addition to more general sociodemographic characteristics such as sex, age and working and household situations [5], psychological determinants such as motivation or behavioural regulation (the reason *why* a person participates in sports) and goals (*what* an individual is expecting to achieve with sports) have been found to be strong intrapersonal determinants of sports participation. Based on self-determination theory (SDT) [17], various studies have found that more self-determined and autonomous types of motivation have an important impact on (persistence in) sports participation [18,19]. Other studies have highlighted the importance of intrinsic goals (e.g., developing skills, seeking challenge, gaining social affiliation and improving health) for participation in sports and physical activity and sports frequency [19].

Recently, some studies have shown that motivations and goals are related not only to sports participation but also to an individual’s choice of a specific sports setting. For instance, Borgers and colleagues [6] found that sports participation in non-traditional settings (including running, cycling and gym activities) seemed to be driven by values related to healthism and physical appearance, whereas members of sports clubs were more likely to practice sports because of sociability or performance-related goals. For participants who participate in gym or outdoor settings under the guidance of a fitness instructor, health management and skill development goals were most important, followed by physique enhancement and social affiliation [20]. A Norwegian study showed that adults who exercise in natural environments reported stronger motives concerning convenience (e.g., easy accessibility in terms of time, location, money and ‘practising at their own pace’) and experiencing nature than did gym or organized sports participants, who reported stronger motives for improving physical health and sociability [9].

While these studies give a first indication of the differences in motivations and goals of users of different sports settings, a systematic comparison of possible interactions between sports settings and level of self-determined motivation and goals and the association with sports participation is currently lacking. Such a comparison would contribute to a better understanding of the variations in preferences and requirements of sports participants across different settings for sports participation. Related to the question of how users of different sports settings differ in motivation and goals is the question of how these differences relate to the frequency of sports participation, which is an important policy outcome indicator [21].

Our study applies a socio-ecological framework, which is frequently used in studies in the health and physical activity domains and recently in studies on sports participation [22,23]. According to the socio-ecological approach, there are multiple influences on specific health behaviours, including factors on the intrapersonal, interpersonal, environmental levels. All influences on behaviours potentially interact across these different levels [24]. Although most socio-ecological models recognize the existence of interactions between factors at multiple levels, they often do not offer clear hypotheses on how these factors interact [25]. Because the current literature shows mixed empirical evidence for individual-environmental interactions in explaining physical activity or sports participation, as results differ greatly depending on the specific interactions studied, more research is needed regarding the interactions of different socio-ecological levels for specific health behaviours [25]. As interactions between motivations and goals, which are important psychological determinants of sports participation, and sports settings, which are environmental determinants, have not yet been studied in relation to sports participation, this study fills this gap.

Based on the social-ecological framework, we hypothesize that both sociodemographic and motivational variables may have different effects on sports frequency depending on the specific setting for sports activities. For instance, users of informal settings such as public spaces are more flexible regarding the times they want to practice sports, compared to participants in more traditional sports settings with fixed time schedules. Therefore, it is possible that participants in informal self-organized sports settings (such as public spaces) might need a higher level of autonomous motivation, and are driven by different (intrinsic) goals than participants in traditional sports clubs to participate frequently. For instance, their sports frequency could be fostered trough autonomy and flexibility. For participation in the more social settings of traditional sports clubs, it is hypothesized that social goals and commitment help trigger the (autonomous) motivation to participate in sports frequently. In addition, the fixed trainings, competitions and obligations or expectations from coaches and peers might stimulate their extrinsic goals and therefore sports frequency. For participants in gyms or health centres, it is more difficult to anticipate what role motivations and goals impact sports frequency. More insight into these mechanisms may help in determining what strategies may be useful to further promote sports participation among users of different settings.

In light of the above, the present study aims to investigate 1) how users of different settings differ regarding self-determined motivations and goals, and sociodemographic and sports-related characteristics and 2) how the association of motivations and goals with sports frequency may differ between users of different sports settings.

## Methods

### Study design and respondents

Data were collected via an online survey that recorded information about motivations, goal content, and sports participation characteristics, including principal sports setting. Data collection occurred in six municipalities in the Netherlands (Amsterdam, Utrecht, Alphen aan den Rijn, Heerlen, Berkelland and Roerdalen) in September 2014. These municipalities were selected based on their differences in population density to yield sufficient variation in the availability and accessibility of sports activities and facilities. Eighteen thousand adults (3,000 per municipality), aged 18–80 years old, were randomly selected from municipal population registers. They were invited to participate in the study by their municipality, by means of an official letter by post. This letter contained the link and unique credentials for the online survey. In total, 1,663 respondents completed the survey (9.2% response rate). We have excluded the following respondents from the analyses: those who did not participate in sports or who participated less than once a month (N = 477), those who participated in an inactive form of sports (e.g., bridge) (N = 20), and respondents with missing sociodemographic data (N = 256). The final sample included 910 participants. The total study sample (N = 1,663) was not fully representative for the Dutch adult population due to a underrepresentation of low-educated respondents (12.1% compared to 33% nationally [26]), and of respondents with a non-native Dutch origin (10.8% compared to 21.4% nationally [27]). However, these issues did not lead to an overrepresentation of the share of sports participants in the sample, as 70% of our sample participated three times or more in sports per month, which is similar to the percentage of sports participants among the general Dutch population [28]. This suggests that a selection bias towards more sports-minded respondents has not occurred. In addition, participants of our sample used similar sports settings than the general adult population [28]. In addition, the correction for education level in our multivariate analyses implies that the results represent the general relation across education levels in a reliable way. Furthermore, due to the cross-sectional design of the study, the directions of the associations found is unknown and do not imply causality.

Ethical guidelines were followed although ethical approval was not required according to the Ethics Committee of Utrecht University.

### Measures

In the survey, respondents were asked to note their principal type of sports, that is, the sport in which they participated most frequently during the 12 months prior to the survey. Subsequently, they were asked in what location that sports activity mostly occurred (referred to as *sports location*, which includes a traditional—often voluntary run—sports club, a registered—often commercially run—sports facility, or a public space) and their *organizational setting* (that is, whether they participated as a member of a traditional sports club; as a participant of a gym, health centre or sports facility other than a sports club; or as part of an informal group or individually). Sports participation was defined as ‘purposeful active participation in sports related physical activities performed during leisure-time’ [10,29,30]. Survey questions on sports participation, sports location and organizational setting were derived from the standardized and validated Dutch guidelines for sports participation research [31,32]. All variables that relate to sports participation (including frequency, setting, motivations, goals, and type of sports) refer to the respondents’ participation in their principal type of sports.

#### Sports frequency

Sports frequency was measured as a self-reported categorical variable with 4 categories: 1 to 3 times a month, once a week, twice a week and at least 3 times a week.

#### Sports setting

Based on survey questions about the sports location and organizational setting that were used most often for participation in the principal type of sports over the past year (see above), the variable *sports setting* was composed. Sports setting was categorized into three groups: 1) *club-organized settings*: users of official sports club facilities, as members of sports clubs, 2) *non-club organized settings*: users of facilities such as gyms, health centres or swimming pools, without traditional club membership, and 3) *informal (public space) settings*: users of (mostly) public spaces practising sports in an unorganized or informal way (e.g., individually, with a friend, or in a small group). According to Borgers et al. [10], club-organized sport refers to participation in a conventional–often voluntary run–association that offers sports activities based on formal membership agreements. Non-club-organized sports entails all other forms of participation outside of a club, which generally takes place in organizational settings, such as self-organized participation in informal groups or alone, but also in commercial health and fitness centres, alternative programmes and facilities offered by municipal sport services or company-based sport [10,29,30]. In contrast to Borgers et al. [10], we consider non-club organized sports in gyms, health centres or swimming pools as a distinct category, because municipal policies regarding these more commercial sports suppliers differ from sports clubs and public space settings.

#### Motivation for sports participation

The 15-item Behavioural Regulation in Exercise Questionnaire (BREQ) [33], which is based on SDT, was translated into Dutch and used to investigate intrinsic motivation and identified, introjected and external exercise-based motivational regulations. Participants responded to the question ‘Why did you participate in your principal sport during the past 12 months?’. Items included, for instance, ‘I participate in sports because people say I should’ for external regulation and ‘It's important to me to exercise regularly’ for identified regulation. Each item was rated on a 5-point Likert scale ranging from 1 (totally disagree) to 5 (totally agree). Principal components analysis (PCA) revealed a slightly different factor structure compared to the theoretical division. We removed the item ‘I get restless if I don’t participate in my sport regularly’ because reliability analysis indicated that the internal consistency of the introjected regulation subscale was too low if we included this item. Other studies show similar measurement issues with the same item [34]. For the items that remained, we calculated mean scores per factor derived from the PCA. The internal consistency of the BREQ subscales was as follows: intrinsic motivation (α = 0.89), identified regulation (α = 0.67), introjected regulation (α = 0.75) and external regulation (α = 0.82). Based on previous research [35,36], scores from the BREQ were used to create variables representing controlled and autonomous motivation. Controlled motivation (α = 0.85) was calculated by obtaining the average from the extrinsic subscales (external and introjected regulation). Autonomous motivation (α = 0.81) was calculated by obtaining the average of the identified and intrinsic regulation subscales.

#### Goals for sports participation

The 20-item SDT-based Goal Content for Exercise Questionnaire (GCEQ) [37] was translated into Dutch and used to assess the importance that participants attribute to intrinsic goals (i.e., skill development, social affiliation, and health management) and extrinsic goals (i.e., image and social recognition) with regard to sports participation. Participants responded to the question ‘Why do you participate in your sport?’ and rated the extent to which the goals were important for participation in their principal sport during the past year on a 5-point Likert scale ranging from 1 (totally disagree) to 5 (totally agree). The factor structure resulting from the PCA corresponded with the original classification. To ensure consistency with the sports motivation measure, we decided to use the mean scores of the factors instead of the factor scores derived from the PCA. The internal consistency of the five subscales was as follows: skill development (α = 0.90), social affiliation (α = 0.88), health management (α = 0.80), image (α = 0.89) and social recognition (α = 0.88).

#### Potential confounders

We controlled for the following demographic characteristics in the multivariate analyses: age, sex, and education. Education was classified into three levels based on the highest self-reported level of completed education: 1) lower education (i.e., no education, primary education, and lower professional education), 2) middle education (i.e., intermediate and higher general education), and 3) higher education (i.e., higher professional education and university). Individual (net) income level was excluded because of the large share of respondents (N = 197) that answered, ‘don’t know/I prefer not to mention’. In addition, we controlled for neighbourhood density level because the sports settings used and the participation rates could differ between urban and rural areas [38,39]. This measure was based on the number of addresses within a radius of one square kilometre from the home location [27] and was aggregated to a 4-digit postal code level. Three categories of address density were distinguished: rural (< 500 addresses per km^2^), hardly to moderately urbanized (500–1.500 addresses per km^2^), and strongly to extremely urbanized (> 1.500 per km^2^).

Health and sports related potential confounders included perceived health, BMI, type of athlete and type of sports. Both perceived health and BMI were controlled for because because they possibly could be related to our independent and dependent variables [21,40]. Perceived health refers to how respondents described their physical health and was classified in three categories: (very) bad to moderate, good and very good. Body mass index (BMI) was calculated based on self-reported height and weight and categorized into underweight to normal weight (< 25), overweight (25–30), and obese (> 30). Type of athlete was self-reported and gives an indication of the level of experience and competitiveness in sports and consists of four categories: 1) those who do not know how to classify themselves as ‘type of athlete’ 2) novice recreational athletes, 3) experienced recreational athletes, and 4) competitive athletes who participate in competitions, matches or races.

### Statistical analyses

SPSS 24.0 was used to provide descriptive statistics on respondents’ personal, motivational and sports participation characteristics. Chi-squares and analyses of variance (ANOVA) were conducted to test for significant differences between participants of the three different sports settings (i.e., those mainly using sports clubs, non-club organized, or informal (public space) settings) regarding their motivations and goals for sports participation and other characteristics (sociodemographic and sports-related characteristics). Furthermore, multivariate ordinal regression analyses were performed to investigate how sports frequency (outcome variable) was determined by motivations, goals, and the use of sports settings, controlled for confounders. To test whether the association of motivations and goals with sports frequency differs between sports settings, interactions between types of motivations and sports settings and interactions between types of goals and sports settings were included.

## Results

### Descriptive results

Descriptive results are presented in Table 1. The mean age was 50.6 (SD = 15.8), and 55.1% of respondents were women. Most respondents engaged rather frequently in sports; 59.1% participated at least twice a week in their principal sport, and this percentage increased to 68.1% if all other sports activities were also included. Individual types of sports were most popular (70.1%), including working out individually in a gym (19.3%), running (13.2%) and types of cycling (11.6%). Most participants indicated that unorganized informal settings (mainly a public space) were their principal sports setting (55.4%), followed by sports clubs (26.3%) and non-club organized settings (facilities such as gyms) (18.4%). Most participants described themselves as an experienced recreational athlete (58.7%). Participants scored relatively high on autonomous motivation (mean score 4.1 out of 5; SD = 0.6) and health management goals (3.9; SD = 0.7), followed by image (3.0; SD = 1.0) and skill development goals (2.9; SD = 1.1).

**Table 1:** Personal characteristics of respondents using different sports settings.

	Total (N = 910)	Club-organized settings (N = 239)	Non-club organized settings (e.g., gyms) (N = 167)	Informal settings (e.g., public space) (N = 504)	P-values
*Age, mean (SD)*	50.6 (15.8)	48.2 (17.3)	48.3 (15.01)	52.5 (15.0)	0.000
*Female (%)*	55.1	51	70.1	52	0.000
*Education (%)*					
Low Middle High	12.1 36.2 51.8	9.2 38.9 51.9	8.4 41.3 50.3	14.7 33.1 52.2	0.051
*Neighbourhood density (%)*					
Rural Hardly–moderately urbanized Strongly–extremely urbanized	30.7 31.2 38	38.1 31.4 30.5	24.6 34.7 40.7	29.2 30.2 40.7	0.015
*Perceived health (%)*					
(Very) bad–moderate Good Very good	23.6 63.3 13.1	14.6 66.9 18.4	21 66.5 12.6	28.8 60.5 10.7	0.000
*BMI (%)*					
Under–healthy weight (BMI < 25) Overweight (BMI 25–30) Obese (BMI > 30)	58.9 33.5 7.6	57.7 39.3 2.9	62.3 27.5 10.2	58.3 32.7 8.9	0.007
*Sports frequency (main sport) (%)*					
1–3 times a month Once a week Twice a week At least 3 times a week	9.5 31.3 32 27.3	5.9 32.6 29.7 31.8	6.6 32.3 39.5 21.6	12.1 30.4 30.6 27	0.012
*Sports location (%)*					
Indoor sports facility Outdoor sports facility Swimming pool Public space	38.4 15.3 6.6 39.7	35.6 48.9 5.3 10.2	94.1 0.7 1.3 3.9	19.6 2.4 9.2 68.8	0.000
*Organisational setting (%)*					
Organized–sports club member Organized–in gym/health centre Unorganized/informal–with others Unorganized/informal–individually	26.3 18.4 30.3 25.1	100 - - -	- 100 - -	- - 54.8 45.2	0.000
*Type of sports (%)*					
Individual sports Team sports	70.1 29.9	37.7 62.3	61.7 38.3	88.3 11.7	0.000
*Type of athlete (%)*					
Don’t know Novice recreational athlete Experienced recreational athlete Competitive athlete	10.4 18.6 58.7 12.3	4.2 7.9 49 38.9	10.8 25.1 62.9 1.2	13.3 21.4 61.9 3.4	0.000
*Self-determined motivation (mean, SD)*					
Autonomous motivation Controlled motivation	4.1 (0.6) 1.6 (0.6)	4.2 (0.5) 1.6 (0.7)	4.1 (0.6) 1.6 (0.7)	4.1 (0.7) 1.6 (0.6)	0.007 0.3
*Goal content (mean, SD)*					
Skill development Social affiliation Health management Image Social recognition	2.9 (1.1) 2.6 (1.0) 3.9 (0.7) 3.0 (1.0) 1.9 (0.8)	3.6 (0.9) 3.3 (0.8) 3.8 (0.7) 2.8 (0.9) 2.1 (0.8)	2.9 (1.0) 2.4 (0.9) 4.1 (0.6) 3.4 (0.9) 1.9 (0.8)	2.7 (1.0) 2.4 (1.0) 4.0 (0.7) 3.0 (1.0) 1.8 (0.8)	0.000 0.000 0.000 0.000 0.000

### Differences between users of different sports settings

The results of descriptive analyses, Chi-squares and ANOVA analyses are presented in Table 1 and show that significant differences exist in personal characteristics between users of different sports settings.

Compared to users of other settings, members of sports clubs more often lived in rural areas (38.1%) and perceived their health as very good (18.4%), and a relatively large number of them participated in sports very frequently (at least 3 times a week) (31.8%). Most of them perceived themselves as competitive athletes (38.9%) and participated in team sports (62.3%) with ball sports and racket sports as the largest categories. The goals of sports club participants were relatively often related to social affiliation (M = 3.8; SD = 0.8), skill development (M = 3.6; SD = 0.9), and social recognition (M = 2.1; SD = 0.8).

Participants of non-club organized settings such as gyms and health centres were most frequently women (70.1%). Most of these sports participants participated twice a week in sports (39.5%) and engaged in individual sports activities in gyms or in exercise or dance classes.

Informal sports participants more frequently perceived their health as (very) bad to moderate than did users of other settings. These informal sports participants mostly used public spaces as their sports location (68.8%) and were diverse regarding their sports frequency. In particular, individual types of sports such as running, types of (race) cycling, and gym activities were practised.

Participants in non-club organized and informally in public spaces more frequently identified themselves as recreational athletes, whether novice or experienced: 88% in non-club organized and 83.3% in informal settings, compared to 56.9% in sports clubs. Participants in these settings reported both relatively high scores in on health management (M = 4.1; SD = 0.6 for non-club organized, M = 4.0; SD = 0.7 for informal/public space participants) and image goals (M = 3.4; SD = 0.9 for non-club organized, M = 3.0; SD = 1.0 for informal participants) as well.

#### Associations of motivations, goals, and sports settings with sports frequency

Table 2 shows the results of ordinal logistic regressions in which motivations, goals and the use of a certain sports setting were related to sports frequency. The first model (Nagelkerke R^2^ = 0.173) showed the main effects of sports settings, motivations, goals, and confounders. In the second model (Nagelkerke R^2^ = 0.183), interaction effects between motivations and sports setting were added to model 1. In the third model (Nagelkerke R^2^ = 0.212), interaction effects between goals and sports setting were added to model 2.

**Table 2:** Ordinal regression analyses on sports frequency, with interaction effects of motivations and goals with sports setting.

	Model 1 (main effects)		Model 2 (interaction effects with motivations)		Model 3 (interaction effects with goals)	
	Estimate	SE	Estimate	SE	Estimate	SE
*Threshold parameters*						
*Sports frequency (at least twice a week = ref)*						
1–3 times a month Once a week Twice a week	-0.95 1.14 2.68	0.67 0.67 0.67	-1.23 0.88 2.43	0.78 0.78 0.79	-0.89 1.25 2.84	0.81 0.81 0.82
*Confounders*						
*Age*	0.02**	0.01	0.02**	0.01	0.02**	0.01
*Sex (male = ref)*	-0.05	0.13	-0.06	0.13	-0.06	0.14
*Education (high = ref)*						
Low Middle	-0.25 0.11	0.21 0.14	-0.26 0.08	0.21 0.14	-0.23 0.09	0.21 0.14
*Neighbourhood density (strongly-extremely = ref)*						
Rural Hardly–moderately	0.02 0.40*	0.16 0.16	0.02 0.42**	0.16 0.16	0.02 0.44**	0.16 0.16
*Perceived health (very good = ref)*						
(Very) bad–moderate Good	-0.78** -0.73**	0.23 0.20	-0.76** -0.71**	0.23 0.20	-0.89** -0.81**	0.24 0.20
*BMI (obese = ref)*						
Under–healthy weight Overweight	-0.13 -0.40	0.25 0.25	-0.18 -0.47	0.25 0.26	-0.23 -0.49	0.25 0.26
*Type of sports (team sports = ref)*						
Individual sports	0.80**	0.16	0.83**	0.17	0.84**	0.17
*Type of athlete (competitive athlete = ref)*						
Don’t know Novice recreational athlete Experienced recreational athlete	-1.46** -1.94** -1.43**	0.32 0.29 0.24	-1.53** -1.98** -1.50**	0.32 0.29 0.24	-1.48** -1.91** -1.40**	0.32 0.29 0.25
*Main effects sports setting (informal settings (e.g., public space) = ref)*						
Club-organized settings Non club-organized settings (e.g., gym, health centre)	0.16 0.19	0.19 0.18	0.46 -0.64	1.19 1.29	0.70 -0.11	1.25 1.44
*Main effects motivation*						
Autonomous motivation Controlled motivation	0.41** -0.28*	0.12 0.12	0.44** -0.48**	0.14 0.15	0.45** -0.46**	0.15 0.17
*Main effects goal content*						
Skill development Social affiliation Health management Image Social recognition	0.05 -0.11 0.10 0.16 0.09	0.08 0.09 0.12 0.09 0.11	0.06 -0.10 0.12 0.14 0.09	0.08 0.09 0.12 0.09 0.11	0.00 -0.22 0.37* -0.06 0.26	0.11 0.12 0.15 0.10 0.16
Interaction effects motivation * sports setting (informal settings = ref)						
Autonomous motivation * club-organized Autonomous motivation * non club-organized Controlled motivation * club-organized Controlled motivation * non club-organized			-0.35 0.11 0.72** 0.24	0.26 0.27 0.26 0.26	-0.49 0.03 0.57 0.14	0.31 0.32 0.30 0.33
Interaction effects goals * sports setting (informal settings = ref)						
Skill development * club-organized Skill development * non club-organized Social affiliation * club-organized Social affiliation * non club-organized Health management * club-organized Health management * non club-organized Image * club-organized Image * non club-organized Social recognition * club-organized Social recognition * non club-organized					0.40* -0.02 0.47* 0.24 -0.70* -0.58 0.44* 0.75** -0.33 -0.35	0.18 0.22 0.22 0.26 0.28 0.33 0.21 0.23 0.26 0.30
Nagelkerke R^2^ P model Pearson Chi-Square (P-value)	0.173 0.000 2688.62 (p = 0.579)		0.183 0.000 2685.578 (p = 0.574)		0.212 0.000 2722.75 (p = 0.325)	
*Test of Parallel Lines*						
-2 Log Likelihood (general) Chi-square P-value	2135.368 81.261 0.001		2113.535 93.090 0.001		2065.735 111.461 0.003	

*p < .05

**p < .01.

In all models, respondents with stronger autonomous motivations participated more frequently in sports, and those with stronger controlled motivations participated less frequently in sports. Only the third model showed that goals were associated with sports frequency. It showed that participants with strong health management goals participated more frequently in sports. In none of the models was sports setting directly associated with sports frequency.

### Associations of interactions of motivations and goals with sports setting on sports frequency

Several significant interaction effects of motivations and goals with sports settings were found (Table 2). The second model (including interactions between motivations and sports settings) showed that those participating in club-organized settings with strong controlled motivations had a higher sports frequency. The third model (including interactions between goals and sports settings) indicated that having skill development goals led to a higher sports frequency among sports club members. Social affiliation goals were associated with a higher sports frequency in club-organized and non club-organized settings. Image goals had stronger positive association with sports frequency among participants in non-club organized settings and club-organized settings than among participants in informal settings such as the public space. Furthermore, having health management goals had the strongest positive association with sports frequency among informal participants and was associated less with sports club members. When the interaction effects of goals with sports settings appeared in model 3, the positive relation of controlled motivation on the sports frequency of club members (model 2) disappeared.

## Discussion

In general, this study showed that different sports settings attract different types of sports participants with different levels of self-determined motivations and goals. Goals were particularly highly interrelated with sports settings and impacted sports frequency.

The results of descriptive analyses revealed that sports participants using different settings for their sports practices differed regarding their preferred type of sports and whether the participants were novice, experienced or competitive athletes. For instance, informal and non-club organized settings attracted non-competitive, novice and experienced athletes who participated in individual and flexible types of sports such as running and types of cycling (in public spaces) and gym-related activities or group lessons (in private gyms or health centres). Members of traditional sports clubs, on the other hand, were more experienced and competitive athletes and participated more frequently in team sports. Similar findings were also found in the study of Borgers et al. [5]. Interestingly, the different sports settings also attracted participants with different perceived health statuses, with informal (e.g., public space) participants in general reporting poorer perceived health compared to club members. This finding is in line with the previous literature showing evidence for better psychological and health outcomes in club-based (team) sports participants than individual participants and those in less social settings [21,41]. However, sports participation in outdoor settings can also produce higher restorative health benefits than do indoor settings [42]. In addition, it might be that informal and flexible settings and types of sports that are practised in gyms and public spaces have a lower threshold for people who have (physical) health problems or are overweight, as heavy weight might function as a barrier to joining a sports club [40].

With regard to motivations and goals, descriptive analyses showed that users of informal and non club-organized sports settings were more similar to each other than to sports club members. In accordance with Borgers et al. [6], we found that social goals were mostly found among members of traditional sports clubs. Interestingly, sports club members showed higher levels of both extrinsic goals (social recognition and image) and intrinsic goals (skill development and social affiliation). Although social recognition and social affiliation goals differ from each other, both types of goals are focussed on social relationships with peers and/or coaches. Previous research has shown that these factors are important determinants of participation and continuation in organized sports [43,44]. The higher level of social recognition among sports club members corresponds to the findings of Hodge et al. [45]., who found relative high scores on social recognition and extrinsic levels of motivation among Master athletes in sports clubs (aged 29–77 years), which could be explained by their high ego-orientation (that is, their focus on personal success) in sports. We found a strong association between the goals related to skill development and sports club participants, which might be related to the type of sports (technical level, team sports). Furthermore, in accordance with previous studies [6,9,20], we found that sports participants with health-related goals were primarily found in the more flexible, and/or non club-organized settings such as gyms and public spaces and less in club-organized settings. In general, health improvement goals (such as increasing energy level, stamina, or resistance to illness and disease) were the most prevalent goals for participation in sports among the sample. This could be related to the increased focus on healthy lifestyles and the current ‘healthism’ discourse in Western societies, within which sport is seen to provide a means to be ‘fit’ and to achieve a slim body [4,6,46–48]. Apparently, health goals seem to be related to individual settings and less to traditional organized settings such as sports clubs and competitive types of sports and participants. This implies that traditional sports clubs function to a lesser extent as health-oriented sporting environments. For example, if sports participants perceive the culture within sports clubs as focused on skill development, social recognition and performance and as a place where trainers and peers have expectations and limits are pushed, for instance, this might explain why novice athletes prefer more low-key, flexible opportunities with less sense of obligations [4,8]. In addition, a (perceived) lack of skills necessary to join a sports club might also hinder novice and non-sports participants to become a member of a sports club.

The results of the ordinal regression analyses showed that motivations, goals and interactions of motivations and goals with sports settings were related to sports frequency. In line with SDT-based research [17–19], we found that a higher score on self-determined autonomous motivations was associated with a higher sports frequency, whereas controlled motivations were associated with a lower sports frequency. However, the interaction effects showed that having strong controlled motivations was related to a higher sports frequency particularly among sports club members, in contrast to those in informal (mainly) public space settings. Additionally, the extrinsic goal of image was found to be associated with a higher sports frequency in sports clubs and gym participants. On the other hand, the results revealed that having (intrinsic) skill development and social affiliation goals were associated with a higher sports frequency among sports club members than among non-club organized and informal sports participants. Apparently, traditional sports clubs attract sports participants who want to improve themselves or master their sports techniques. On the other hand, despite strong controlled motivations and extrinsic goals of social recognition and image, club members participate very frequently and spend more time in sports [5]. While less self-determined or controlled motivations and goals theoretically are associated negatively with sports participation [17] and with earlier stages of behaviour change for exercise [49], more serious or competitive athletes might perceive these more extrinsic goals or motivations differently and be motivated to participate more frequently. In addition, the positive associations of social affiliation and skill development goals with sports frequency among club-organized settings and among users of non club-organized settings such as gyms and health centres implies that the social, fun and learning aspects of sport have positive associations with sports participation regardless of the sports setting [21].

Finally, we found that having health management goals had the strongest positive association with sports frequency among participants in informal settings compared to sports club members. Because sports participation in informal settings such as public spaces is often not subject to specific schedules and obligations to others and is free of charge, external triggers to go practise sports are largely lacking. This could be a reason why more individual goals related to one’s own health are needed to decide whether or not to practice. However, more extrinsic socially constructed goals related to ‘healthism’ such as losing weight and improving appearance might also stimulate participants to exercise more frequently.

### Strengths, limitations and future research directions

Strengths of this study includes the fact that we collected sports participation data on different socio-ecological levels, which allowed us to investigate the association of interactions between several motivational variables and specific sports settings on sports frequency. Moreover, we measured both motivations and goals, and these scales were both based on psychological theories of motivation.

Limitations of this study are the low response rate (9.2%) and a sample that consisted of a relative active older age group, whereas respondents with low income and non-Dutch migration background were underrepresented. However, as the sports settings used, and the sports frequency in our sample corresponded to the statistics regarding the general Dutch adult population [28], and because we controlled for relevant intrapersonal variables, a selection bias towards more sports-minded respondents is unlikely.

Future research should consider whether adults participate in more than one type of sport and/or using multiple sports settings, as this might be associated with motivations, goals and sports frequency. Moreover, for sports and health promotion purposes, it is interesting to compare the results with the motivations, goals and barriers related to the use of specific sports settings of non-participants as potential new sports participants. Person-oriented, qualitative research approaches could contribute to this.

## Conclusions and practical implications

Although ample evidence exists about the importance of psychological determinants including motivations and goals for sports as well as environmental determinants for sports participation, little is known about how the relation of motivations and goals with sports frequency differs between users of different sports settings.

The results of this study suggest that different settings for sports participation attract different types of sports participants. They differ in personal characteristics and in their levels of self-determined motivations and goals. Club-organized sports settings were associated with participants who were focussed on intrinsic goals related to skill development and social affiliation and on extrinsic goals related to social recognition from others and image. Users of non club-organized settings (i.e., gyms, health centres and swimming pools) and informal settings (i.e., mainly the public space) were more similar to each other than to sports club members and were associated with individual types of sports and with goals related to image and health improvement, respectively.

Moreover, the results showed that goals in particular were highly interrelated with the choice of a certain sports setting and had impact on sports frequency. Our results indicate that sports frequency is higher when participants engage in settings that are more suitable for their motivations and goals and whether these are more or less self-determined. We noticed that sports clubs, which are usually known for their higher sports frequencies and time spent on sports [5], attracted participants with intrinsic and extrinsic oriented goals. In addition, those with health goals participated more frequently in sports when practising in informal settings such as the public space.

Our findings show evidence for interactions of different socio-ecological levels to explain the complex behaviour of sports participation [24]. While factors of the physical environment are often taken into account as determinants influencing health behaviour, including sports participation [14,50], we recommend also considering interactions on different levels, including psychological-environmental interactions, in research on explaining sports participation.

### Implications

Based on the findings of this study, we recommend policymakers and managers in the sport and health domains to be aware of the increasing importance of health goals and flexible, informal settings among the growing group of recreationally orientated sports participants [3,6,51]. To maintain or increase the number of members and to not lose ground to informal sports settings, sports clubs could offer extra (low threshold, few skills needed) trainings focused on less experienced or less competitive participants and those with poorer health status, who prefer to have more flexibility and less obligation or recognition from others. Moreover, creating a healthy, welcoming and inclusive environment might allow those with more vulnerable health status to feel more at ease at sports clubs [52]. Furthermore, the results suggest an increased attention to making public spaces more attractive and suitable for sports participation. Policymakers could investigate the motivations that different groups of (potential) public space participants have for sports participation and for the use of specific locations. Practically, this can for instance be done by a (qualitative) investigation of what type of spaces sports participants actually use (where are they located, which environmental features do they have, what is the infrastructure like, what types of sports are people practising, whether sports participants interact with each other etc.,), and asking them why they prefer that type of public places, if they are missing something and what improvements they would suggest to make it more encouraging for them to practice sports in the public space.

## Supporting information

S1 File Dataset used for this study. Dataset based on data collection in six municipalities in the Netherlands (2014).
(XLSX)
